# Predicting Cancer Cell Line Dependencies From the Protein Expression Data of Reverse-Phase Protein Arrays

**DOI:** 10.1200/CCI.19.00144

**Published:** 2020-04-24

**Authors:** Mei-Ju May Chen, Jun Li, Gordon B. Mills, Han Liang

**Affiliations:** ^1^ Department of Bioinformatics and Computational Biology, The University of Texas MD Anderson Cancer Center, Houston, TX; ^2^ Department of Cell, Development and Cancer Biology, Knight Cancer Institute, Oregon Health & Science University, Portland, OR; ^3^ Department of Systems Biology, The University of Texas MD Anderson Cancer Center, Houston, TX

## Abstract

**PURPOSE:**

Predicting cancer dependencies from molecular data can help stratify patients and identify novel therapeutic targets. Recently available data on large-scale cancer cell line dependency allow a systematic assessment of the predictive power of diverse molecular features; however, the protein expression data have not been rigorously evaluated. By using the protein expression data generated by reverse-phase protein arrays, we aimed to assess their predictive power in identifying cancer dependencies and to develop a related analytic tool for community use.

**MATERIALS AND METHODS:**

By using a machine learning schema, we conducted an analysis of feature importance based on cancer dependency and multiomic data from the DepMap and Cancer Cell Line Encyclopedia projects. We assessed the consistency of cancer dependency data between CRISPR/Cas9 and short hairpin RNA–mediated perturbation platforms. For a fair comparison, we focused on a set of genes with robust dependency data and four available expression-related features (copy number alteration, DNA methylation, messenger RNA expression, and protein expression) and performed the same-gene predictions of the cancer dependency using different molecular features.

**RESULTS:**

For the genes surveyed, we observed that the protein expression data contained substantial predictive power for cancer dependencies, and they were the best predictive feature for the CRISPR/Cas9-based dependency data. We also developed a user-friendly protein-dependency analytic module and integrated it with The Cancer Proteome Atlas; this module allows researchers to explore and analyze our results intuitively.

**CONCLUSION:**

This study provides a systematic assessment for predicting cancer dependencies of cell lines from different expression-related features of a gene. Our results suggest that protein expression data are a highly valuable information resource for understanding tumor vulnerabilities and identifying therapeutic opportunities.

## INTRODUCTION

Understanding the genotype-phenotype relationships of cancer cells is a central task for precision cancer medicine because it will help classify patients into different treatment groups and identify novel therapeutic targets. The recent genome-wide short hairpin RNA (shRNA) or CRISPR/Cas9-mediated cell viability screens provide a unique opportunity to systematically characterize cancer dependencies in human cancer cell lines.^1-3^ For example, the DepMap portal has curated the dependency profiles of approximately 18,000 genes across more than 500 human cell lines. Several studies have assessed the possibility of predicting cancer dependency from genomic or transcriptomic features.^3,4^ Although proteins are basic functional units in most biologic processes and represent the vast majority of therapeutic targets, proteomic features have not been evaluated along with those DNA- or RNA-level features in such studies.

**CONTEXT**

**Key Objective**
This study aimed to systematically assess the predictive power of different expression-related features of a gene for its cancer dependency through a rigorous machine learning (ML)–based feature importance analysis and develop the related bioinformatics module for community use.
**Knowledge Generated**
Reverse-phase protein array (RPPA)-based protein expression data contain substantial predictive power as messenger RNA (mRNA) expression for cancer dependencies. Through our newly developed analytic module, researchers can discover novel genotype-phenotype patterns, generate testable hypotheses, and interpret biologic findings in a tumor context–dependent manner.
**Relevance**
This is a systematic analysis that assesses the predictive power of protein expression in inferring gene dependencies across a large number of cell lines. The developed analytic module is a valuable informatics tool for understanding tumor vulnerabilities and identifying therapeutic opportunities.

RPPAs are a powerful approach to generate functional proteomics data. This quantitative antibody-based assay can assess a large number of protein markers in many samples in a cost-effective, sensitive, and high-throughput manner.^5-7^ By using RPPAs, we have characterized a large number of patient and cell line samples through The Cancer Genome Atlas,^8,9^ Cancer Cell Line Encyclopedia (CCLE),^10-13^ and MD Anderson Cell Line projects.^14^ Furthermore, we have built an open-access, dedicated bioinformatics resource, The Cancer Proteome Atlas (TCPA), for the cancer research community to study these large-scale functional proteomic data in a rich context.^14-17^ Here, we used a rigorous machine learning (ML) schema to evaluate the cancer-dependency predictive power of the RPPA-based protein expression along with other expression-related molecular features (ie, copy number alteration [CNA], DNA methylation, and mRNA expression). We also implemented a new protein-dependency analytic module in TCPA, thereby allowing users to explore, analyze, and visualize the relationships between protein expression and cancer dependency.

## MATERIALS AND METHODS

### Collection of RPPA, Cancer Dependency, and Other Molecular Profiling Data

We downloaded the RPPA data from the CCLE,^10-13^ which assayed 214 protein markers across 899 cell lines (https://portals.broadinstitute.org/ccle). We obtained cancer dependency data, including CRISPR/Cas9 (DepMap19Q1)^2,18^ and shRNA (DEMETER2)^1^ data sets, from the DepMap portal (https://depmap.org/portal). We also collected CNA, DNA methylation, and mRNA expression data from CCLE (https://portals.broadinstitute.org/ccle).

### Model Outcome and Feature Engineering

**FIG 1.**
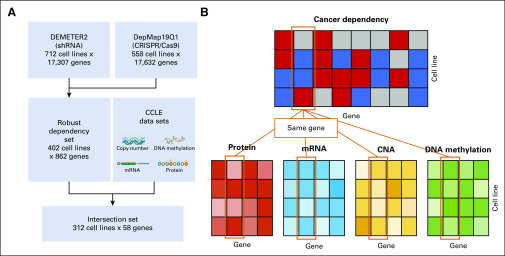
Data processing and the definition of the machine learning problem. (A) The procedure to obtain the final set that contains the data of the model outcome and all available features. (B) A cartoon representation of the cis-prediction (same gene) of cancer dependency from the four expression-related features, including protein (orange), messenger RNA (mRNA; blue), copy number alteration (CNA; yellow), and DNA methylation (green).

We considered a regression task in dependency scores (cell growth change) that experienced gene knockdown (shRNA) or knockout (CRISPR/Cas9). Specifically, the response variable (model outcome) is a vector of dependency scores for each gene across cell lines. A score of 0 indicates that a gene is not essential, whereas a score of –1 corresponds to the median value of all common essential genes. The explanatory variables (predictors) were the self-features that were related to gene expression. To ensure the quality of the model outcome, we first constructed a robust cancer dependency set by collecting genes and cell lines that showed high consistency between shRNA (DEMETER2) and CRISPR/Cas9 (DepMap19Q1) experiments. Next, this robust dependency set was overlapped with the cell lines and genes from CCLE to extract available data for model training (Fig 1A). To conduct a fair comparison, we considered only three expression-related DNA or RNA features (CNA, DNA methylation, and mRNA expression) with the RPPA-based protein expression data (total protein levels) from the same set of cell lines and performed cis-prediction (for the same gene) between the cancer dependency (response variable) and molecular features (explanatory variables) (Fig 1B).

### ML Schema

**FIG 2.**
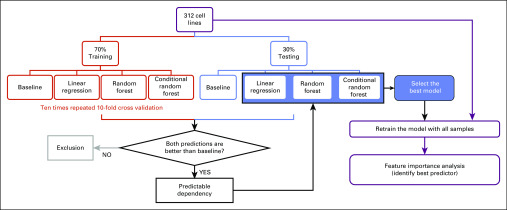
Overview of machine learning schema.

As shown in Figure 2, the samples (cancer cell lines) were randomly split into a training set (70%) and a held-out testing set (30%). To test which ML algorithm performed best, we adopted three common classifiers: linear regression, random forest, and conditional random forest. We also conducted a baseline model to exclude failed predictions by using the averaged dependency score as the predicted values. For model training, we performed 10-fold cross validation using the training set and repeated the procedure 10 times to avoid model overfitting. Then, we applied the trained models on the held-out testing set. The prediction performance was measured and compared using the root-mean-square error (RMSE) and *R*^2^. We trained models for each gene dependency. A dependency was flagged as predictable if it had at least one classifier that outperformed (had lower RMSE than) the baseline model in both the training and testing predictions. For the genes with predictable dependencies, we selected the best classifier (with the highest *R*^2^) based on the testing results and used the selected classifier to retrain the model using all samples. Finally, to evaluate the individual contribution of each feature, we performed a feature importance analysis to identify the best predictor for each dependency. We implemented this ML schema in R v3.5.0 using the caret package^19^ with the ML methods of lm, rf, and cforest. In addition, we used the varImp function to estimate the feature importance.

### Development of the Protein-Dependency Analytic Module

We used R and Python libraries to process and analyze the data. All the precomputed analytic results were converted into the JSON format and loaded into the CouchDB database for users to query and analyze. We used JavaScript D3 and the Angular library to construct the Web user interface of the protein-dependency analytic module. The module displays the table results by DataTables and the nested plots by HighCharts.

### Data Sharing Statement

The data and results are available at the TCPA website (http://tcpaportal.org/mclp).

## RESULTS

### Construction of a Robust Cancer Dependency Data Set

**FIG 3.**
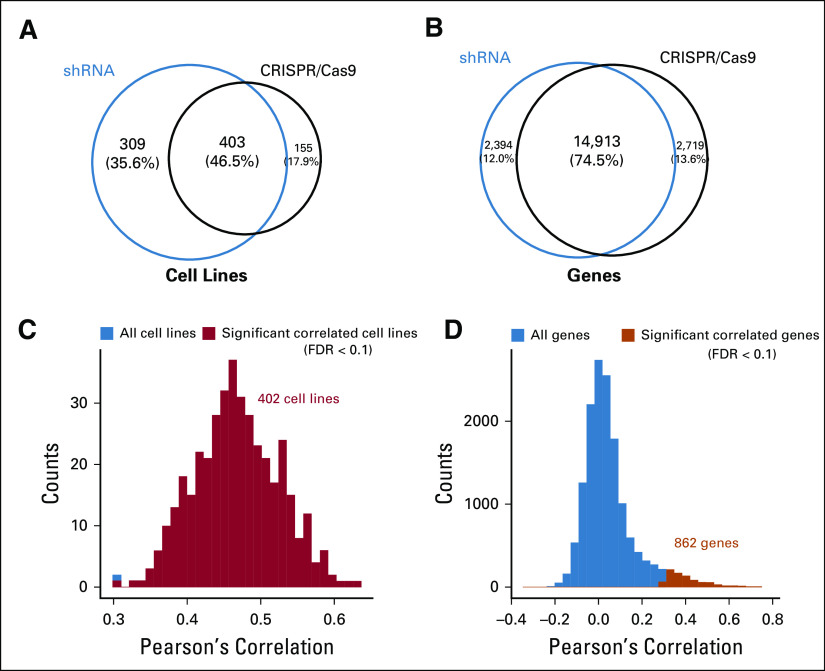
Common dependencies between short hairpin RNA (shRNA) and CRISPR/Cas9 experiments from DepMap. (A) Venn diagram of cancer cell lines from the two sets. (B) Venn diagram of genes from the two sets. (C) Histogram of Pearson’s correlations across all genes for each cancer cell line (sample-wise correlation). (D) Histogram of Pearson’s correlations across cancer cell lines for each gene (gene-wise correlation). The significant correlations (*P* < .01, false discovery rate [FDR] < 0.1, and *R* ≥ 0.3) are highlighted in red (C), and orange (D).

To ensure the data quality of the cancer dependency scores, we constructed a robust cancer dependency set. The shRNA (DEMETER2) and CRISPR/Cas9 (DepMap19Q1) platforms shared 403 cancer cell lines and 14,913 genes (Fig 3A-B). We first evaluated the consistency between the two platforms by computing Pearson’s correlations across genes for each cell line (Fig 3C) and the correlations across cell lines for each gene (Fig 3D). The results showed that almost all the cell lines (99.8%, except for one) showed significant positive correlations of cross-platform cancer dependencies (*P* < .01; false discovery rate [FDR] < 0.1; *R* ≥ 0.3). In contrast, only 862 genes (5.8%) showed significant positive correlations across cell lines (*P* < .01; FDR < 0.1; *R* ≥ 0.3). This pattern suggested that many of the dependencies resulted from random effects and thus could not be preserved across the platforms. In subsequent analyses, we retained only 402 cell lines and 862 genes that showed significant consistency between the shRNA and CRISPR/Cas9 platforms to reduce potential random noise.

### Predictive Power of Protein Expression in Cancer Dependency

**FIG 4.**
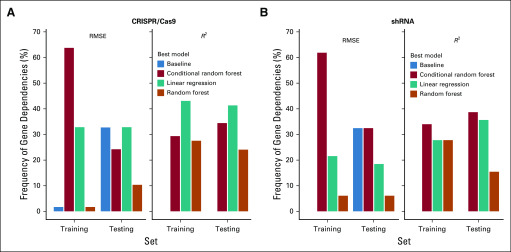
Frequencies of the best models observed in all the tested gene dependencies. For the dependency of a gene, the root-mean-square error (RMSE) or *R*^2^ scores were computed for every model in either the training or the testing set. For each measurement type, the scores from the four models were compared, and a model was selected as the best if it exhibited the best performance (the smallest RMSE or the largest *R*^2^). The bar plots show the counts of the observed best models among all 58 tested dependencies. A dependency was flagged as a failed prediction if the baseline model was selected as the best according to the RMSE score. (A) Bar plots based on the CRISPR/Cas9 platform (34.5% of the dependencies failed). (B) Bar plots based on the short hairpin RNA (shRNA) platform (36.2% of the dependencies failed).

**FIG 5.**
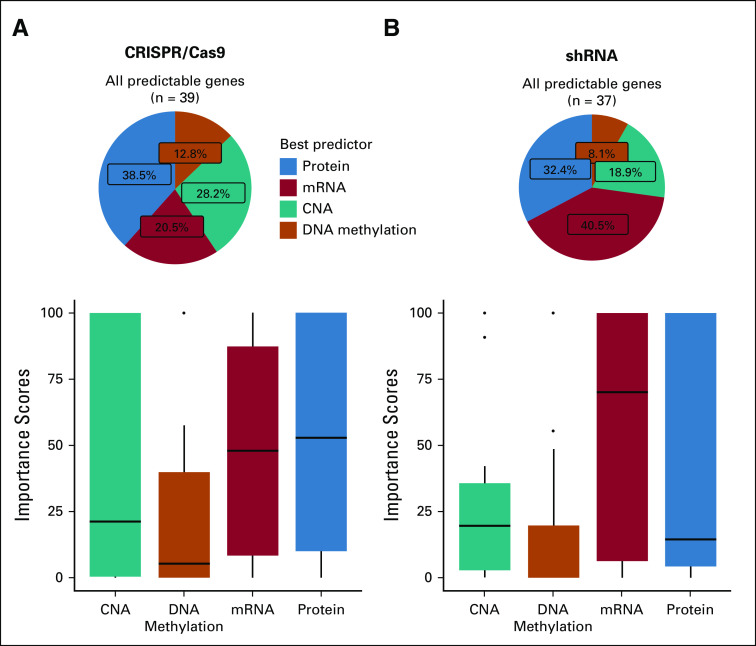
Feature importance analysis among the predictable gene dependencies. We evaluated the feature importance for approximately 40 predictable dependencies using the varImp function in the R package caret. Appendix Figure A1 shows the performance of the selected models used for this importance analysis. The importance scores were normalized to the range of 0% to 100%. For each dependency, we compared the importance scores of the four features and selected the one with the highest score as the best predictor. (A) A pie chart showing the frequency of the observed best predictor and a box plot showing the important score distribution of each feature based on the CRISPR/Cas9 platform. (B) A pie chart showing the frequency of the observed best predictor and a box plot showing the important score distribution of each feature based on the short hairpin RNA (shRNA) platform. CNA, copy number alteration; mRNA, messenger RNA.

On the basis of the designed ML schema (Fig 2), we assessed the predictive power for 58 genes in 312 cell lines for which all four expression-related features (CNA, DNA methylation, mRNA, and protein expression) were available. First, we excluded the unreliable predictions from the assessment. By comparing with the baseline models, we found that more than 60% of gene dependencies could be successfully predicted from self-expression–related features irrespective of the platform (CRISPR/Cas9, 65.52%, Fig 4A; shRNA, 63.79%, Fig 4B). The ML models learned better from the CRISPR/Cas9 platform than from the shRNA platform. Next, among the genes with predictable dependencies, we investigated which feature was the most important in inferring cancer dependencies. As shown in Fig 5A, 38.5% of the dependencies could be best inferred by protein expression when using the CRISPR/Cas9 platform data, followed by CNA (28.2%), mRNA expression (20.5%), and DNA methylation (12.8%). But the pattern changed when using the shRNA-defined dependencies (Fig 5B); the best predictor was mRNA expression (40.5%), followed by protein expression (32.4%), CNA (18.9%), and finally DNA methylation (8.1%). We then investigated the importance score distributions of the four features (Fig 5; Appendix Tables A1 and A2) and found that, in both platforms, the importance of the protein expression feature could not be distinguished from that of the mRNA feature. Nor could the importance of the CNA feature be distinguished from that of the protein and mRNA features in the CRISPR/Cas9-defined dependencies, even though the CRISPR/Cas9 dependencies had been corrected for copy-number effect. The different patterns observed for the shRNA and CRISPR/Cas9-defined dependencies may be a result of certain fundamental technical issues. For example, the superior performance of the mRNA feature in shRNA-defined dependencies is likely caused by the knockdown effects targeting mRNAs. Despite the relatively small number of genes and proteins surveyed, our results suggest that RPPA-based protein expression data contain substantial predictive power for cancer dependencies, at least equivalent to RNA sequencing–based mRNA expression, and they perform best when using CRISPR/Cas9 dependencies.

### Newly Developed Protein-Dependency Analytic Module in TCPA

**FIG 6.**
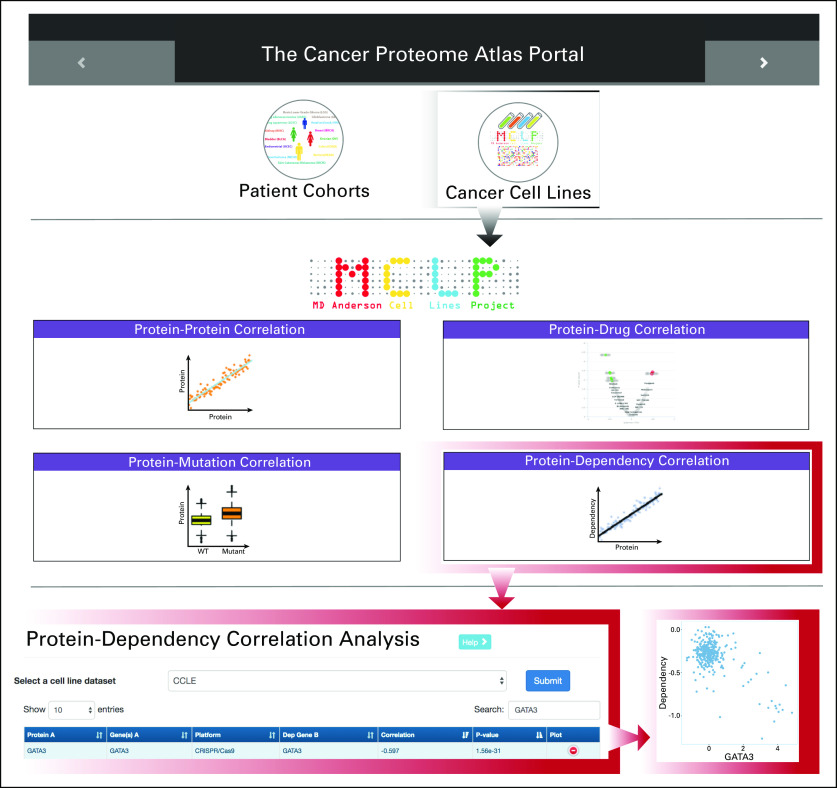
Snapshot of the protein-dependency analytic module in The Cancer Proteome Atlas. The newly added module is highlighted in red boxes.

The above results highlight the utility of RPPA-based proteomic data in understanding cancer phenotypes and identifying novel therapeutic targets. Therefore, we developed a protein-dependency analytic module and integrated it into the cell-line Web platform of TCPA. This user-friendly, interactive module allows researchers to explore, visualize, and analyze the relationships between the RPPA and cancer dependency data. We included two independent RPPA data sets^10,14^ for users to examine the protein-dependency relationships of interest with ease. The module provides a straightforward, intuitive table view so that users can investigate whether the expression level of a protein is a good predictor for the corresponding cancer dependency across cancer cell lines (tested by Pearson’s correlation; visualized by scatter plots; Fig 6). The first column contains the protein markers, followed by the knockdown or knockout genes and their assessment platform (shRNA or CRISPR/Cas9) and then the corresponding statistic and *P* value.

## DISCUSSION

In this study, we assessed the potential of RPPA-based protein expression to infer cancer dependencies through a rigorous ML-based feature importance analysis. To the best of our knowledge, this is the first systematic analysis to elucidate the predictive power of protein expression in inferring gene dependencies across a large number of cell lines. Our findings provide a strong rationale for incorporating protein expression data into the prediction tasks of cancer dependencies. One limitation of this study is the relatively small number of genes and proteins assessed, which limits the statistical power compared with other expression-related features. Our RPPA platform covers only approximately 200 protein markers, and we are in the process of expanding the protein list to approximately 500 proteins. We will revisit this topic when a larger RPPA data set becomes available. In addition to the dependency of its gene, the protein level likely helps predict the effects of other genes, and for such an analysis, a similar ML strategy equipped with advanced feature selection techniques is warranted. We also implemented a new analytic module in TCPA that can be used to directly analyze and visualize the relationships between protein expression and cancer dependencies across cancer cell lines. This module will help researchers discover novel genotype-phenotype patterns, generate testable hypotheses, and interpret biologic findings in a tumor context–dependent manner. We expect it to be a valuable bioinformatics tool for the cancer research community.

## Figures and Tables

**FIG A1.**
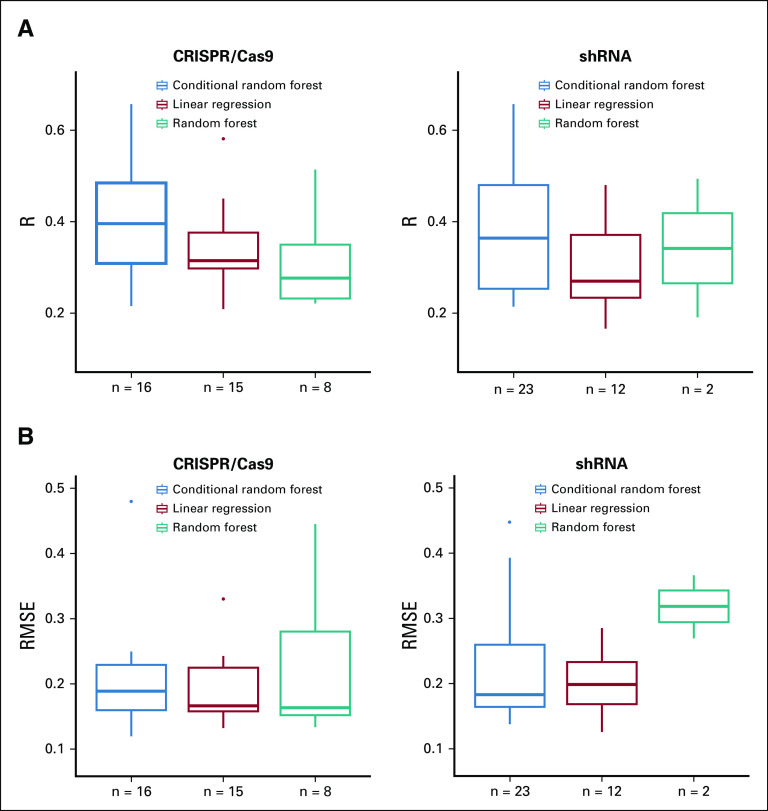
Performance of the selected models in Figure 5. (A) Box plots of *R* (Pearson’s correlations) stratified by model types. (B) Box plots of RMSE stratified by model types. RMSE, root-mean-square error; shRNA, short hairpin RNA.

**TABLE A1.**
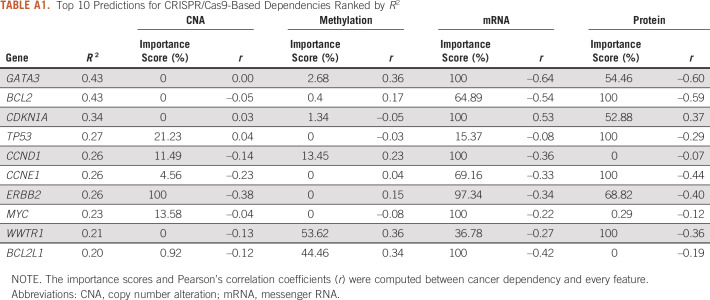
Top 10 Predictions for CRISPR/Cas9-Based Dependencies Ranked by *R*^2^

**TABLE A2.**
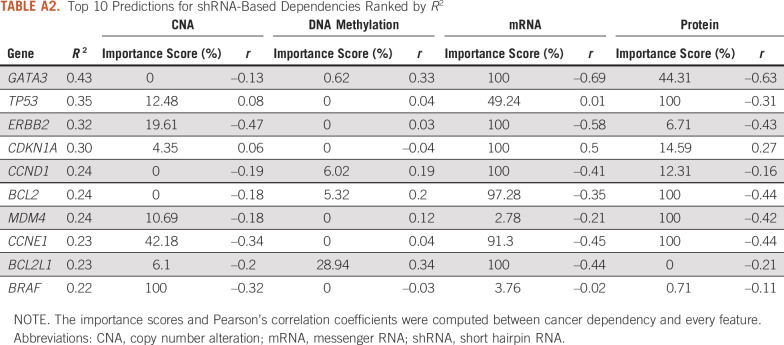
Top 10 Predictions for shRNA-Based Dependencies Ranked by *R*^2^
